# Regional Anesthesia With Levobupivacaine in a Patient With a RyR2 Gene Mutation After Cardiac Arrest: A Case Report

**DOI:** 10.7759/cureus.84874

**Published:** 2025-05-27

**Authors:** Svetlana Sreckovic, Jana Lemic, Katarina Vitomirovic, Petar Vukman, Ivana Glisovic Jovanovic

**Affiliations:** 1 Anesthesiology, University of Belgrade, Belgrade, SRB; 2 Anesthesiology and Reanimation, University Clinical Centre of Serbia, Belgrade, SRB; 3 Orthopedics and Traumatology, University Clinical Centre of Serbia, Belgrade, SRB; 4 Orthopedics, University of Belgrade, Belgrade, SRB

**Keywords:** axillary nerve block, hypoxic/anoxic ischemic encephalopathy, ryr2 gene mutation, ultrasound-guided regional anesthesia, levobupivacaine

## Abstract

Catecholaminergic polymorphic ventricular tachycardia (CPVT) is a rare inherited arrhythmia syndrome affecting the structurally normal heart, occurring during high adrenaline levels triggered by exercise or emotional stress. CPVT results from a mutation in the *RyR2* gene and is clinically characterized by episodes of syncope, arrhythmias, or sudden cardiac arrest. Optimal perioperative preparation for patients with CPVT aims to prevent increases in catecholamine levels during venipuncture, surgery, and pain management. Levobupivacaine, a long-lasting local anesthetic, was administered to a 28-year-old female patient for an axillary nerve block during orthopedic surgery. The patient had experienced sudden cardiac arrest at the age of 24, where the *RyR2* gene mutation was confirmed, leading to the initiation of beta-blocker therapy. Subsequent hypoxic-ischemic encephalopathy, resulting from resuscitation, caused spastic quadriplegia. The patient's vital parameters, such as electrocardiogram, non-invasive blood pressure (NIBP), and oxygen saturation (SpO2), were monitored throughout the perioperative period. Orthopedic surgery was successfully completed, with no changes observed in the electrocardiogram. Levobupivacaine, being less cardiotoxic, ensured good intraoperative conditions without adverse events and provided adequate postoperative pain control for the patient with CPVT during orthopedic surgery.

## Introduction

Insufficient oxygen supply to the brain results in various disabilities that affect muscle tone, motor functions, movement, and posture. Depending on the area of the brain that is affected, the severity, and the duration of oxygen deprivation, spastic quadriplegia can occur [1-3]. Therefore, proper perioperative management of these patients is essential and requires a precise understanding of the etiology, pathophysiology, and clinical implications of spastic quadriplegia. However, patients with spastic quadriplegia can present significant challenges for positioning and administering regional anesthesia. Catecholaminergic polymorphic ventricular tachycardia (CPVT) results from mutations in the *RyR2* gene, leading to spontaneous calcium release from the sarcoplasmic reticulum in cardiac cells, which is characterized by syncopal episodes and sudden cardiac death [4,5].

Verbal informed consent was obtained from the patient, but the consent form was signed by the parent because a spastic deformity prevented the patient from holding the pen and writing.

## Case presentation

The female patient was born full term through natural childbirth to healthy parents, whose early psychomotor development progressed normally. At the age of 24, the patient experienced chest and abdominal pains, followed by a loss of consciousness, breathing difficulties, and sudden cardiac arrest. Resuscitation was initiated immediately by one parent. Five minutes after resuscitation began, the patient was intubated, and her heart rate exceeded 200 beats/minute. Extracardiac and structural heart disease were excluded after examination, and a *RyR2* gene mutation was identified as the cause of the sudden cardiac arrest. Whole exome sequencing revealed a heterozygous missense variant of the* RyR2* gene, similar to that found in one parent. Beta-blocker therapy was initiated immediately (bisoprolol 1.25 mg every 12 hours). Hypoxic-ischemic encephalopathy, resulting from the resuscitation efforts, has led to spastic quadriplegia.

**Figure 1:**
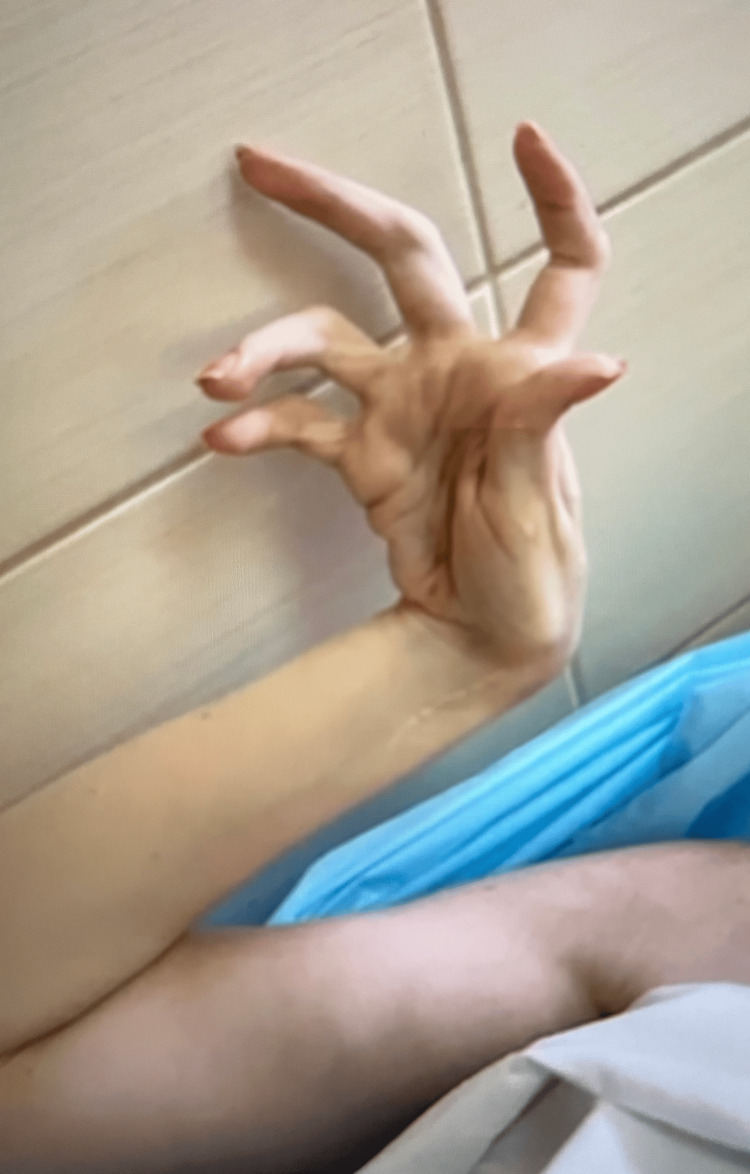
Right hand (preoperatively)

The patient was unable to open her mouth (Mallampati IV). Her hands were primarily positioned in adduction at the shoulder joint and flexion at the elbow joint. Additionally, both hands exhibited significant contractures at the wrist and small joints, taking on a forced semi-flexed position of the hands and fingers (Figure 1).

Bilateral hypertonia from spasticity allowed only limited spontaneous movement in the shoulder joints on both sides, more pronounced on the right. This was accompanied by some flexion and extension movements in the elbow joints, more distinct on the right and minimal on the left, along with restricted, predominantly flexion and extension movements in the hand joints and small joints of the fingers, with no possibility of thumb opposition on either side. Both legs were primarily extended, showing significant contractures and deformities at the feet held in a forced position. Limited flexion was evident in the knee and ankle joints, with no toe movement. Achieving a standing position was impossible.

**Figure 2:**
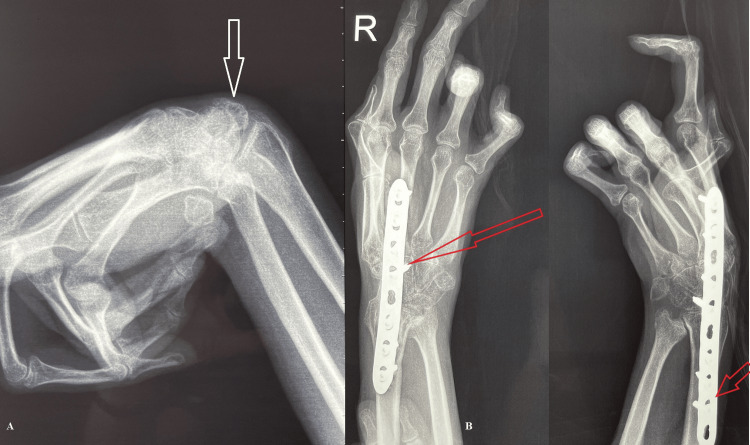
X-ray of the right hand A: Preoperative (white arrow: wrist contracture). B: Postoperative (red arrow: LCP). LCP: locking compression plate

The patient was scheduled to undergo arthrodesis of the right wrist using a locking compression plate (LCP) to enhance hand functionality (Figure 2).

**Figure 3:**
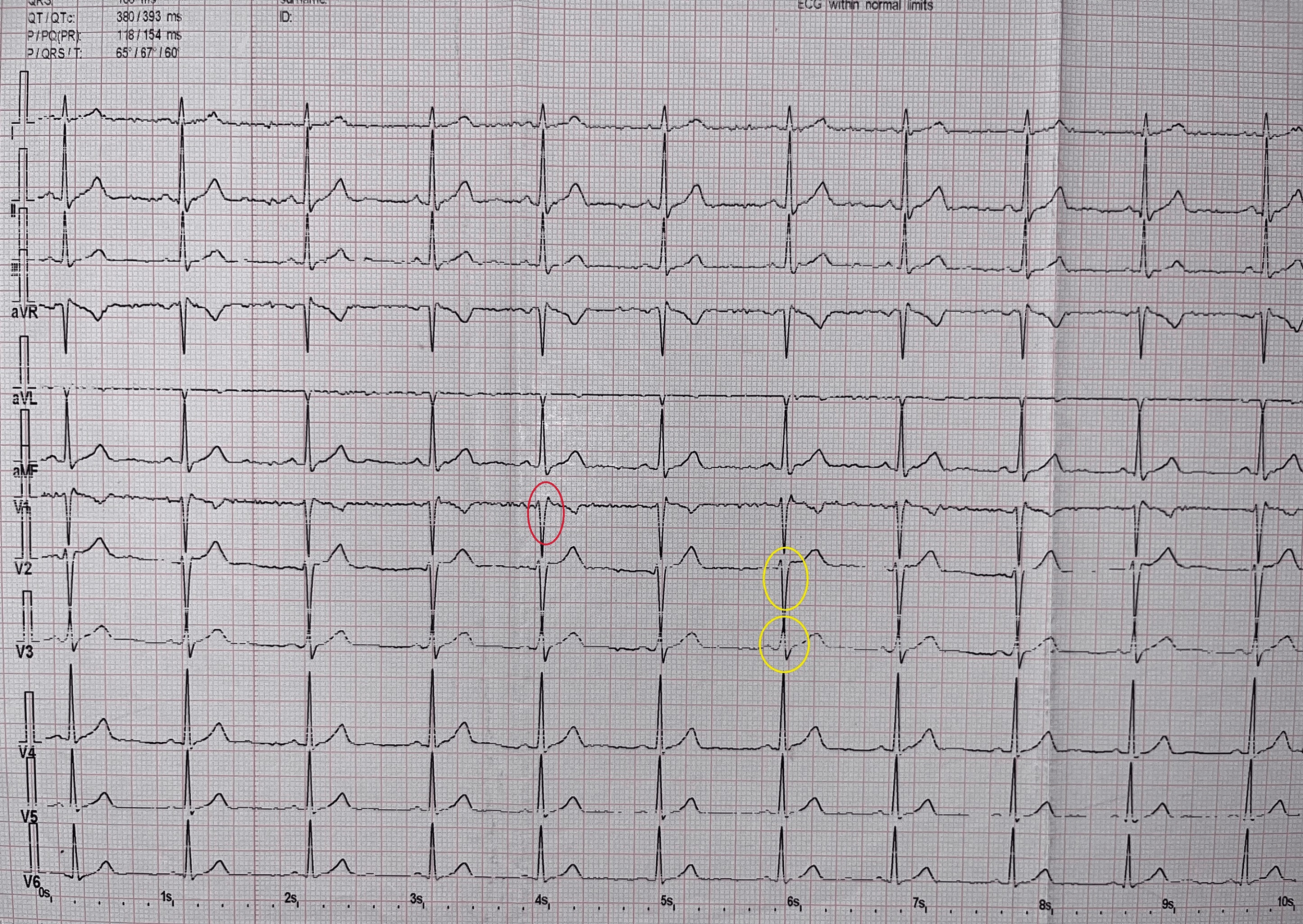
Electrocardiogram preoperatively Red circle: rSr in V1. Yellow circle: rS u in V2 and V3.

A neurologist and a cardiologist examined the patient before surgery. A routine electrocardiogram (Figure 3) and a blood test, which included a blood cell count, biochemical analysis, and coagulation profile, with no abnormalities detected, were also conducted.

**Figure 4:**
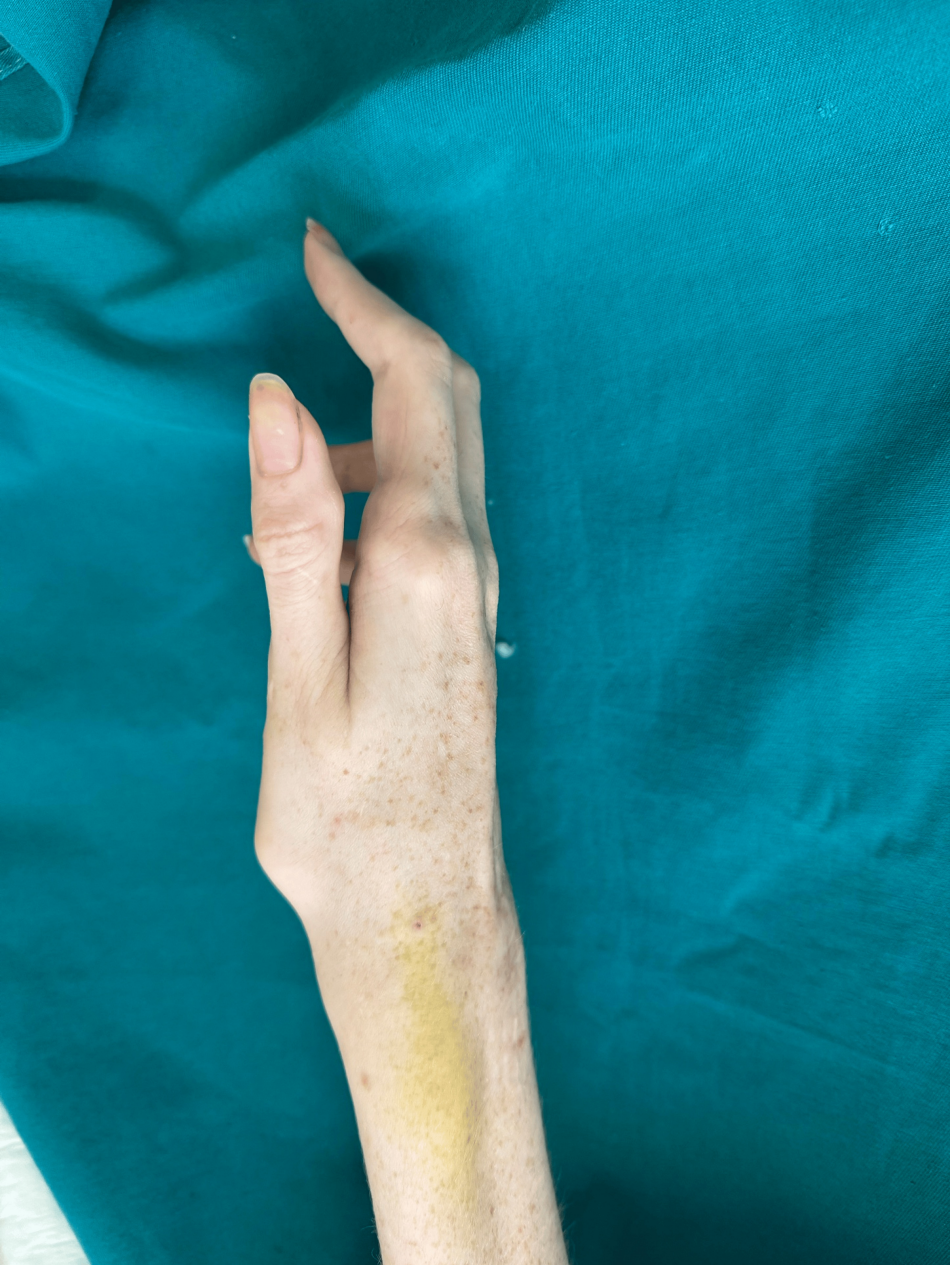
Right hand (postoperatively)

Beta-blocker therapy was continued on the day of surgery. Vital parameters (electrocardiogram, non-invasive blood pressure (NIBP), and saturation (SpO2)) were monitored preoperatively, and during and after surgery. Preoperatively, NIBP was 108/75 mmHg, heart rate was 75 beats/minute, and SpO2 was 100%. Venipuncture was performed using lidocaine gel, with no change in vital parameters noted (103/66 mmHg, heart rate: 76 beats/minute, SpO2: 100%). Premedication with midazolam 2 mg intravenously preceded regional anesthesia. In the lying position, abduction of the right arm was possible up to 60°. Levobupivacaine was used as a local anesthetic, known for its reduced cardiotoxicity. The axillary nerve block was performed using a linear ultrasound probe. After identifying the musculocutaneous, median, ulnar, and radial nerves, 3.5 mL of 0.33% levobupivacaine was injected per nerve using a 22-gauge 50-mm echogenic needle (Stimuplex® insulated, B Braun Medical, Melsungen, Germany). During surgery, midazolam was administered in a bolus dose of 1 mg, resulting in the patient falling asleep (for a total of 2 mg). The onset of motor block occurred 11 minutes after the block was applied. The operation was conducted in a bloodless field using a tourniquet, which was inflated 20 minutes after the block. Surgery commenced at the 30th minute and lasted 95 minutes. Throughout the operation, blood pressure and heart rate remained stable (systolic: 95-110 mmHg, diastolic: 60-75 mmHg, heart rate: 60-67 beats/minute). The patient regained sensation six hours after the block was performed and was discharged home the next day (Figure 4).

## Discussion

CPVT is a rare inherited arrhythmia syndrome that affects structurally normal hearts and occurs during high adrenaline levels, such as during exercise or emotional stress. It is clinically characterized by episodes of syncope, arrhythmias, or sudden cardiac arrest [5,6]. Increasing intracellular calcium levels is not the only mechanism needed to produce the arrhythmia associated with CPVT. Following increased calcium levels, cardiomyocytes can readjust homeostasis and restore sinus rhythm [6]. However, adrenergic stimulation disrupts this autoregulation, particularly in the presence of an *RYR2* gene mutation [6].

The *RyR2* gene mutation is responsible for approximately 70% of CPVT [5,6]. More than 170 missense variants have been identified, but their pathogenic role remains unclear due to the gene's large size and the presence of many rare nucleotide polymorphisms that are family-specific. The primary clinical symptom of CPVT is syncope. The underlying cause of syncope is the onset of polymorphic ventricular tachycardia (VT), which can either resolve spontaneously or lead to sudden cardiac arrest, with mortality rates ranging from 30% to 50% by the age of 20-30 years [5,6].

Treatment of CPVT depends on symptom manifestation and includes medications and lifestyle changes, especially sports restrictions for high-risk patients [5,7,8]. Beta-blocker therapy is recommended for patients who expect adrenergic-dependent trigger activity. The dosage should be individualized, and to prevent the recurrence of syncope, nadolol (1-2.5 mg/kg/day, either in a single dose or divided into two doses per day) or propranolol (2-4 mg/kg/day, divided into 3-4 doses per day) has been used. If syncope recurrence or complex arrhythmias occur during exercise, treatment with flecainide (100-300 mg/day) ± beta-blockers is necessary. An implantable cardioverter-defibrillator and left cardiac sympathetic denervation should be considered for patients with recurrent syncope, polymorphic VT, or cardiac arrest despite optimal medical therapy [5,7,8]. A beta-blocker was initiated after a sudden cardiac arrest, and since then, our patient has experienced no syncope or any other symptoms of CPVT.

The perioperative management of a patient with CPVT aimed to avoid factors that could trigger serious arrhythmias, such as tachycardia, sympathetic stimulation, and the use of beta agonists or sympathomimetic drugs throughout the entire perioperative period. Therefore, mitigating catecholamine peaks caused by fear or insufficient anesthesia/analgesia begins preoperatively, effectively reducing anxiety during venipuncture, surgery, and pain management while minimizing loud noises, voices, or stressful behaviors [6-8]. In this patient, venipuncture was performed using lidocaine gel, and no changes in vital parameters were detected. Furthermore, premedication with benzodiazepines (midazolam) was utilized to alleviate anxiety and facilitate the nerve block. Additionally, the subsequent administration of midazolam during surgery induced sleep.

Although the relevant data are limited, various types of anesthesia have been used in patients with CPVT, and no significant anesthesia-related complications have been reported [9-11]. Adequate intraoperative anesthesia and maintaining oxygenation, normocarbia, normothermia, and normovolemia are crucial. The appropriate type of anesthesia must achieve and maintain deep levels of anesthesia while avoiding hypotension and bradycardia, thus eliminating the need for sympathomimetic drugs. Patients with CPVT often have relatively low or borderline blood pressure, especially those receiving high doses of beta-blockers or calcium channel blockers. General anesthetics can further reduce this, particularly during induction [9-11]. Likewise, combining beta-blockers with anesthetics may induce episodes of bradycardia [9-12].

Propofol is the most commonly used intravenous anesthetic for inducing and maintaining anesthesia in patients with CPVT [9-12]. Thiopental has been linked to increased plasma concentrations of noradrenaline, while data on etomidate's use in CPVT is insufficient; ketamine should be avoided due to its sympathomimetic properties. Among volatile agents, isoflurane and sevoflurane have been used without adverse effects [9-12]. Intraoperative analgesia with fentanyl and alfentanil has been safely achieved for these patients. Non-depolarizing agents have minimal direct effects on cardiac electrophysiology, except for pancuronium, which has vagolytic effects and carries a risk of inducing tachycardia [9-12]. The potential vagal stimulation and potassium shifts explain why succinylcholine is avoided in patients with CPVT [13].

Peripheral nerve blocks, a type of regional anesthesia, have become widely utilized due to their advantages: effective postoperative analgesia, reduced opioid consumption, lower rates of postoperative nausea and vomiting, fewer pulmonary complications, enhanced early rehabilitation, and increased patient satisfaction [14]. Local anesthetics with long-acting properties and improved safety profiles contribute to their increased usage. However, concerns are growing regarding their potential cardiotoxicity and central nervous system (CNS) toxicity. Levobupivacaine and ropivacaine are amide-based, long-acting local anesthetics developed as safer alternatives to bupivacaine, but clinical studies present conflicting results concerning their analgesic and anesthetic characteristics [15]. Li et al., in a meta-analysis, compared the efficacy of ropivacaine and levobupivacaine in peripheral nerve blocks [16]. They found no significant difference between these two local anesthetics regarding onset time for surgical anesthesia, sensory block, motor block, duration of motor block, and overall patient satisfaction. Additionally, they reported that levobupivacaine provided longer anesthesia and better postoperative analgesia. The duration of the sensory block with levobupivacaine is longer than that of ropivacaine [16]. In our patient, postoperative pain intensity reached up to 3 on the Numerical Rating Scale, and there was no need for opioids.

Plakhotnik et al. demonstrated no difference in cardiotoxicity among bupivacaine, ropivacaine, and levobupivacaine at doses lower than 2 μg/mL [17]. The study concentrated on human stem cell-derived cardiomyocytes. It was established that, in addition to NaV1.5 cardiac sodium channel inhibition being the sole mechanism of cardiotoxicity, dysregulation of calcium dynamics also plays a role [17]. Furthermore, bupivacaine influences calcium channels, including the ryanodine receptor. At doses of 3 and 6 μg/mL, bupivacaine disrupts L-type calcium channels and the ryanodine receptor sarcoplasmic calcium efflux, while ropivacaine and levobupivacaine have no effect [17].

In this case, if an acute arrhythmia had occurred during regional anesthesia, it could have indicated local anesthetic systemic toxicity (LAST) or CPVT. Beta-blockers are the primary treatment for acute CPVT [5,6]. Esmolol, owing to its short half-life and intravenous formulation, is recommended as the first choice [5,6]. Furthermore, intravenous flecainide has successfully managed VT. In instances of hemodynamic instability, arrhythmia, or any symptoms of LAST, we prepared Intralipid 20%, phenylephrine, atropine, esmolol, and an external defibrillator.

## Conclusions

Catecholaminergic polymorphic ventricular tachycardia (CPVT) is a rare inherited arrhythmia syndrome that affects structurally normal hearts, leading to syncopal episodes and sudden cardiac death. It results from mutations in the *RyR2* gene, causing spontaneous calcium release from the sarcoplasmic reticulum in cardiac cells. Proper anesthetic management for patients with CPVT requires an understanding of potential risks and careful planning, avoiding factors that might increase catecholamine levels during the perioperative period. Utilizing regional anesthesia with levobupivacaine, which is less cardiotoxic, ensures favorable intraoperative conditions without adverse events and provides effective postoperative pain control for patients with CPVT undergoing orthopedic surgery.
